# Randomized Trial of Antibiotics in Addition to Tocolytic Therapy to Treat Preterm Labor

**DOI:** 10.1155/S106474499400013X

**Published:** 1994

**Authors:** D. Heather Watts, Marijane A. Krohn, Sharon L. Hillier, David A. Eschenbach

**Affiliations:** 1 Department of Obstetrics and Gynecology University of Washington 1959 NE Pacific Street RH-20 Seattle WA 98195 USA; 2 Department of Epidemiology University of Washington Seattle WA USA

## Abstract

*Objective:* The objective of this study was to assess whether antibiotic therapy plus tocolysis given to women in preterm labor would prolong pregnancy compared with tocolysis alone.

*Methods:* A randomized, double-blind trial of intravenous mezlocillin and oral erythromycin therapy vs. placebo was used in addition to tocolysis among women in preterm labor ≤34 weeks gestation with intact membranes. Amniocentesis was performed, and chorioamnionic membranes were examined histologically and cultured for microorganisms after delivery.

*Results:* Clinical characteristics including gestational age at enrollment, frequency of contractions, cervical Bishop's score, and white blood cell count on admission were similar in the 2 groups. Antibiotic therapy was well tolerated. No significant differences in the interval to delivery, birth weight, and neonatal outcomes were observed between the 2 groups. Women in the antibiotic group had a significantly lower incidence of postpartum infections compared with women in the placebo group. Patients with evidence of upper genital tract infection in either group had a significantly shorter interval to delivery, lower gestational age at delivery, lower mean birth weight, and increased neonatal hospitalization time.

*Conclusions:* Lack of an antibiotic effect on the gestational age at delivery may be due to the low prevalence of upper genital tract infection among unselected women in preterm labor, to advanced preterm labor unresponsive to antibiotic therapy, or to an inability of antibiotics given alone to inhibit the cytokine response. Further work is needed to identify markers of upper genital tract infection among women in preterm labor and to evaluate other potential therapeutic interventions.


Infectious Diseases in Obstetrics and Gynecology 1:220–227 (1994)
© 1994 Wiley-Liss, Inc.

### Randomized Trial of Antibiotics in Addition to Tocolytic Therapy to Treat Preterm Labor

**D. Heather Watts, Marijane A. Krohn, Sharon L. Hillier, and David A. Eschenbach**

*Departments of Obstetrics and Gynecology (D.H.W., M.A.K., S.L.H., D.A.E.) and Epidemiology (D.A.E.), University of Washington, Seattle, WA*

### Abstract

***Objective:*** **The objective of this study was to assess whether antibiotic therapy plus tocolysis given to women in preterm labor would prolong pregnancy compared with tocolysis alone.**

***Methods:*** **A randomized, double-blind trial of intravenous mezlocillin and oral erythromycin therapy vs. placebo was used in addition to tocolysis among women in preterm labor ≤34 weeks gestation with intact membranes. Amniocentesis was performed, and chorioamnionic membranes were examined histologically and cultured for microorganisms after delivery.**

***Results:*** **Clinical characteristics including gestational age at enrollment, frequency of contractions, cervical Bishop's score, and white blood cell count on admission were similar in the 2 groups. Antibiotic therapy was well tolerated. No significant differences in the interval to delivery, birth weight, and neonatal outcomes were observed between the 2 groups. Women in the antibiotic group had a significantly lower incidence of postpartum infections compared with women in the placebo group. Patients with evidence of upper genital tract infection in either group had a significantly shorter interval to delivery, lower gestational age at delivery, lower mean birth weight, and increased neonatal hospitalization time.**

***Conclusions:*** **Lack of an antibiotic effect on the gestational age at delivery may be due to the low prevalence of upper genital tract infection among unselected women in preterm labor, to advanced preterm labor unresponsive to antibiotic therapy, or to an inability of antibiotics given alone to inhibit the cytokine response. Further work is needed to identify markers of upper genital tract infection among women in preterm labor and to evaluate other potential therapeutic interventions.**
**© 1994 Wiley-Liss, Inc.**

Key words

Antibiotics, prematurity, amniotic fluid infection

Preterm birth remains a major unsolved problem in obstetrics and infection has been suspected to play a role in some preterm deliveries. Upper genital tract (amniotic fluid and chorioamnion) infection has been identified among a higher proportion of women delivering preterm compared with those delivering at term. Amniotic fluid (AF) infection is present in up to ⅓ women presenting in preterm labor (PTL) below 28 weeks gestation.[1] Chorioamnion infection decreases from a rate of 80% below 26 weeks to approximately 10% at term,[2] and histologic chorioamnionitis decreases rapidly from a rate of 55% below 28 weeks gestation to 5% from 29 weeks gestation to term.[3] Thus, the prevalence of upper genital tract infection and inflammation is inversely related to gestational age, and infection appears to contribute to preterm birth in patients with PTL resistant to tocolysis.

**Address correspondence/reprint requests to Dr. D. Heather Watts, Department of Obstetrics and Gynecology, University of Washington, 1959 NE Pacific Street, RH-20, Seattle, WA 98195.**
**Presented at the annual meeting of the Infectious Disease Society for Obstetrics and Gynecology, Seattle, WA, August 1990.**

Received June 14, 1993
Accepted March 7, 1994

Clinical Study

Despite the strong association between upper genital tract infection and preterm delivery, a cause-and-effect relationship between the 2 has not been established. Evidence of a cause-and-effect relationship would be strengthened if a reduction of preterm delivery occurred among infected patients given antibiotics in a randomized double-blind fashion. To evaluate the potential contribution of infection to preterm birth, we randomized women in PTL with intact membranes to antibiotics or placebo in addition to tocolytic therapy. Attempts were made to enroll patients at high risk for infection and to examine the results in the infected vs. the uninfected group.

### SUBJECTS AND METHODS

Women presenting with preterm contractions with intact membranes between October 1986 and October 1987 were evaluated for eligibility for this study. We attempted to enroll patients at increased risk for infection (<34 weeks getation), but without clinical evidence of infection (<38°C temperature), those with no known cause of labor, yet a high chance of actually being in PTL. To be included, women had to be between the ages of 15–45 years, have a singleton gestation without known maternal uterine or fetal anomalies, an estimated gestational age ≤34 weeks, intact membranes, an admission temperature <38°C, contractions occurring at least every 10 min with a documented cervical change, and a Bishop's score of at least 4 with at least 1 cm dilatation or 50% effacement. Women allergic to penicillin or erythromycin or on antibiotics within the previous 7 days, with cervical dilatation >4 cm, with placenta previa, vaginal bleeding more than bloody show, coagulation abnormalities or placental abruption noted on ultrasound, contraindications to tocolytic therapy, or difficulty in communication were excluded. During the study period, 718 women presented to the University of Washington Medical Center with possible PTL and were screened for enrollment. Six hundred ten patients (85%) were excluded: patients with gestational age above 34 weeks, 13%; preterm rupture of membranes, 29%; no labor, 20%; fetal or uterine anomalies, 5%; placenta previa or abruption, 7%; penicillin or erythromycin allergies, 4%; multiple gestation, 9%; recent antibiotic use, 3%; cervix dilated more than 4 cm, 3%; maternal medical conditions, 3%; temperature ≥38°C, 1%; and other conditions including non-English speaking or psychotic, 3%. In addition, 29 women refused participation. Of the remaining 79 women who were eligible for enrollment, 56 were approached and signed informed consent to participate in the study.

All women underwent ultrasound for assessment of gestational age and fetal health. Women with adequate AF volume who consented underwent amniocentesis for fetal lung maturity studies and microbiologic cultures. Aliquots of AF were transported in a syringe to the clinical microbiology laboratory for routine bacterial culture and transported immediately to the research microbiology laboratory or placed into a Port-a-Cul vial (Becton-Dickinson Co., Inc., Cockeysville, MD) where 100 μl of AF was cultured as previously reported.[1] Urine was obtained by bladder catheterization for urinalysis and culture. A speculum examination was performed to collect vaginal specimens and to exclude rupture of the fetal membranes. A vaginal swab was rolled onto a clear glass slide and observed microscopically for a ferning pattern to rule out ruptured membranes. The pH of the vaginal fluid was determined by pH paper. Vaginal fluid was also collected on cotton swabs for Gram stain and transported in Amies transport medium (Medical Media Lab, Boring, OR) for cultures of group B *Streptococcus* and *Escherichia coli* as previously described.[4] Cervical specimens were obtained on dacron swabs for Gram smear and inoculated onto Thayer-Martin media for *Neisseria gonorrhoeae* cultures and centrifuged onto McCoy cell monolayers for *Chlamydia trachomatis* cultures as previously indicated.[5] Vaginal and cervical smears were stained with crystal violet and Gram's iodine with a saffranin counterstain and evaluated for the presence of white blood cells (WBCs) and bacterial vaginosis according to published criteria.[6] Bishop's score was determined by digital examination of the cervix.

After obtaining vaginal AF, urine, and cervical specimens for culture, we randomly assigned the women in a blinded fashion to either 1 of 2 groups: 1) mezlocillin, 3 g intravenously (IV) every 6 h, and erythromycin ethylsuccinate, 333 mg orally every 8 h; or 2) identical placebos of each. The antibiotic regimen was chosen to inhibit the most common microorganisms isolated from AF and the chorioamnion isolates at our hospital, including anaerobic and facultative bacteria and *Ureaplasma*

*urealyticum*.[1,2,7] IV therapy was continued for 5 days and oral therapy for 10 days. Tocolytic therapy was administered to all women. The choice of tocolytics was at the discretion of the resident and attending physician staff and included IV ritodrine or magnesium sulfate, intramuscular ritodrine, and/or oral indomethacin. Women whose PTL had initially been stopped on parenteral tocolytic therapy received oral terbutaline or ritodrine until delivery or 36 completed weeks gestation. Bethamethasone in standard doses was administered at the discretion of the attending and resident physician staff. Women at less than 34 weeks gestation with recurrent episodes of contractions were retreated with parenteral tocolytics unless the cervix was more than 4 cm dilated or the membranes were ruptured.

Maternal obstetrical, laboratory, and delivery data were collected by interview and chart review. At delivery, the chorioamnion was cultured and examined histologically as previously described.[2] Infant outcomes were recorded by a combination of chart review and interview of the mother. Respiratory distress syndrome was diagnosed in the presence of 1) tachypnea and intercostal or substernal retractions, 2) the need for supplemental inspired oxygen to maintain arterial oxygen pressure greater than 50 torr, and 3) bilateral diffuse air bronchograms on chest radiograph. Confirmed neonatal sepsis was diagnosed in the presence of a positive blood or cerebrospinal fluid culture from the infant within 3 days of delivery.

Categorical variables were compared using the chi-square test with Yates correction or two-tailed Fisher exact test as appropriate. Continuous variables that were normally distributed were compared using the Student's t-test. Continuous variables that were not normally distributed were compared using a median test.[8] Proportional hazard models developed by Cox were used to compare the rates (hazard) of preterm delivery among women receiving antibiotics and those receiving placebo.[9] For the proportional hazards model, the time-to-event was the number of days from enrollment until the patient delivered or reached 35 completed gestational weeks; the outcome event was delivery before 35 completed weeks. The rate (the frequency of outcome events per unit time) of preterm delivery was determined by a computer algorithm that includes, at any given time point, only those women still at risk for preterm delivery. Likelihood ratio tests were used to verify the 95% confidence intervals (CI) for the risk ratio. Kaplan-Meier curves were performed to display the probability of remaining undelivered for women receiving antibiotics or placebo.[10]

### RESULTS

Fifty-six women were enrolled in the study. Thirty were randomized to receive mezlocillin and erythromycin and 26 were randomized to receive placebo. Characteristics of women in each of the groups on admission are summarized in Table 1. The women in the 2 groups did not differ significantly in any of the characteristics listed in Table 1. Almost twice as many women in the antibiotic vs. the placebo group had C-reactive protein (CRP) levels above 1.5 mg/dl, the level previously shown to be elevated in our hospital,[11] but this difference was not statistically significant. AF cultures were positive at enrollment in 4 of 21 women randomized to receive antibiotics and 1 of 15 women randomized to placebo, but only 64% of women underwent amniocentesis before randomization.

Results of genital cultures were similar between the 2 groups. One patient in the antibiotic group had *N. gonorrhoeae* and 1 patient in the placebo group had *C. trachomatis* in the cervix. Group B *Streptococcus* was isolated from 3 (11%) of 28 patients in the antibiotic group and 2 (8%) of 24 in the placebo group. *E. coli* was present in 1 (4%) of 24 patients in the antibiotic group and 0 of 22 patients in the placebo group. No patients in the antibiotic group and 2 patients in the placebo group had $\geqslant 10^5$ bacteria/ml of urine. Vaginal Gram stain results in the antibiotic group were: normal—12 (43%) of 28, intermediate—13 (46%), and bacterial vaginosis—3 (11%). Vaginal Gram stain results in the placebo group were: normal—12 (46%) of 26, intermediate—9 (35%), and bacterial vaginosis—5 (19%).

Women in both groups received similar numbers of doses of study medication A summary of drug dosing and side effects is listed in Table 2. Antibiotic therapy was well tolerated. Nausea and vomiting were as common in the placebo group as the antibiotic group and were likely related to tocolytic and other multiple medications used among women in PTL. The increased diarrhea among women receiving antibiotics may have been related

**TABLE 1. Enrollment characteristics among women randomized to receive mezlocillin/erythromycin or placebo**

| | Mezlocillin/erythromycin (n = 30) | Placebo (n = 26) | P |
|---|---|---|---|
| Maternal age, mean ± SD (years) | 25.9 ± 4.8 | 23.9 ± 5.6 | 0.20 |
| Ethnicity: Caucasian | 23 (77%) | 19 (73%) | 0.76 |
| Currently married | 17 (57%) | 14 (54%) | 0.95 |
| Previous preterm birth/total with previous pregnancy >20 weeks | 9/18 (50%) | 7/14 (50%) | 0.72 |
| Current smoker | 12/26 (47%) | 10/24 (42%) | 0.80 |
| Drug use this pregnancy (excludes alcohol) | 5/27 (19%) | 5/24 (21%) | 0.99 |
| Gestational age, mean ± SD (weeks) | 30.1 ± 3.1 | 30.1 ± 3.2 | 0.98 |
| Gestational age, median (weeks) | 31.0 | 31.25 | 0.95 |
| Bishop's score, mean ± SD | 7.0 ± 2.3 | 6.3 ± 2.7 | 0.30 |
| Cervical dilatation, mean ± SD (cm) | 2.1 ± 1.6 | 2.1 ± 1.2 | 0.95 |
| Positive amniotic fluid culture | 5/22 (23%) | 1/15 (7%) | 0.37 |
| Peripheral WBC count, mean ± SD (1,000/cm$^3$) | 12.3 ± 7.1 | 11.6 ± 5.5 | 0.70 |
| Maximum temperature before enrollment, mean ± SD (°C) | 36.8 ± 4.4 | 36.9 ± 5.1 | 0.50 |
| CRP, median (mg/dl) | 1.75 | 1.45 | 0.30 |
| >1.5 mg/dl | 12/19 (63%) | 6/16 (38%) | 0.20 |
| Bacterial vaginosis on Gram stain | 3/28 (11%) | 5/26 (19%) | 0.50 |

**TABLE 2. Comparison of drug dosing, reason for study drug discontinuation, and side effects between the randomized treatment groups[a]**

| | Mezlocillin/erythromycin (n = 30) | | Placebo (n = 26) | |
|---|---|---|---|---|
| | IV | Oral | IV | Oral |
| No. doses received, mean ± SD | 11.9 ± 7.0 | 15.5 ± 12.3 | 11.3 ± 7.4 | 16.2 ± 12.7 |
| Stopped drug before course completed | 23 | 19 | 17 | 16 |
| Reason for discontinuation | | | | |
| Delivered | 13 | 12 | 10 | 11 |
| Refused further IV therapy | 0 | | 2 | |
| Discharged undelivered | 6 | 1 | 5 | 2 |
| Required other antibodies | 1 | 1 | 0 | 0 |
| Experienced side effects[b] | 3 | 5 | 0 | 3 |
| Nausea/vomiting | | 2 | | 3 |
| Diarrhea | | 4 | | 1 |
| Rash | | 1 | | 0 |
| Total with side effects | | 7 (20%) | | 4 (12%) |

[a]There are no significant differences in side effects or number of doses between the 2 groups.
[b]One patient in each group had more than one side effect.

to either antibiotic. None of the women who developed diarrhea had a positive stool culture for *Clostridium difficile* or a positive *C. difficile* toxin. No significantly different rates of adverse effects related to antibiotics were seen in infants.

Outcome characteristics between the 2 groups in intent-to-treat analyses are shown in Table 3. Women receiving antibiotics did not differ from placebo-treated women in the median gestation at delivery, median birth weight, or median numbers of days from enrollment to delivery. Kaplan-Meier plots of interval from enrollment to delivery are shown in Figure 1. The hazard ratio for preterm delivery in the antibiotic compared with the placebo group is 0.76; 95% CI = 0.37–1.5. Women receiving antibiotics were less likely to develop postpartum infections or to need antibiotic treatment during or after labor compared with women in the placebo group (3/30 vs. 10/26, $P = 0.03$). Positive membrane cultures were present in 7 (32%) of 22 patients in the antibiotic and 9 (45%) of 20 patients in the placebo group (N.S.). The proportions with histologic chorioamnionitis did not differ between the 2 treatment groups (Table 3).

Infant outcomes are compared between the 2 treatment groups in Table 3. Gestational age esti-

**TABLE 3. Pregnancy and infant outcome according to treatment group**

| | Mezlocillin/erythromycin (n = 30) | Placebo (n = 26) | *P* |
|---|---|---|---|
| Gestational age at delivery (weeks) | | | |
| Obstetrical dates | | | |
| Mean ± SD | 33.1 ± 4.8 | 33.5 ± 4.1 | 0.8 |
| Median | 33.8 | 33 | 0.9 |
| Dubowitz score | | | |
| Mean | 34.2 ± 5.0 | 34.8 ± 4.1 | 0.7 |
| Median | 35 | 34.5 | 0.9 |
| Birth weight, mean ± SD (g) | 2,202.4 ± 851.3 | 2,212 ± 862.2 | .97 |
| Birth weight, median (g) | 2,360 | 2,280 | 0.9 |
| Median interval from enrollment to delivery (days) | 15.5 | 15 | 0.8 |
| No. undelivered >1 week | 17 (57%) | 13 (50%) | 0.8 |
| Maternal non-study antibiotic therapy | | | |
| Before delivery | 3 | 5 | 0.4 |
| After delivery | 1 | 6 | 0.04 |
| Total | 3 | 10 | 0.03 |
| Histologic chorioamnionitis | 17/25 (68%) | 14/20 (70%) | 0.9 |
| Infant hospital days | | | |
| Median[a] | 9 | 12 | 0.8 |
| <10 | 16 | 11 | 0.4[b] |
| 10–60 | 8 | 11 | |
| >60 | 5 | 3 | |
| Infant respiratory distress syndrome | 13 | 8/25 | 0.5 |
| Infant antibiotic therapy | 14 | 9/25 | 0.6 |
| 5-min Apgar score, mean ± SD | 7.4 ± 2.3 | 7.2 ± 2.8 | 0.8 |

[a]Does not include one infant who died <1 day of age.
[b]Chi-square for trend.

mates by Dubowitz and birth weights in the 2 groups were similar. The median hospital stay of the infant, need for antibiotic or oxygen therapy, incidence of respiratory distress syndrome, and proportion of infants with prolonged hospital stays did not differ between the 2 groups. Fourteen infants in the group whose mothers were randomized to antibiotics received antibiotics during their hospital stay. One infant was treated with a single dose of penicillin because of a positive maternal culture for *N. gonorrhoeae* at delivery. The remainder received empiric courses of gentamicin and ampicillin after admission to the neonatal intensive care unit because of respiratory distress which could not be differentiated from pneumonia. One infant in this group also received later treatment for a positive culture for coagulase-negative *Staphylococcus* related to an IV catheter infection. In the placebo group, 9 infants received empiric courses of antibiotics. In addition, 1 infant received 2 courses of antibiotic therapy for coagulase-negative *Staphylococcus* bacteremia related to IV catheter infections. No infants had complications related to maternal antibiotic therapy. One infant in each group died. In the antibiotic group, an infant weighing 1,015 g died at less than 1 day of age of respiratory insufficiency related to prematurity. In the placebo group, an infant weighing 1,096 g at birth died on day 79 of life of respiratory complications from prematurity.

Because antibiotics were not expected to have an effect on pregnancies without evidence of infection, outcomes were compared by therapy among subsets of women with or without the potential markers of upper genital tract infection. No difference in pregnancy outcome was found between antibiotic and placebo groups among women with or without bacteria in the AF, WBCs in the AF, CRP levels above 1.5 mg/dl, positive membrane cultures, or histologic chorioamnionitis (data not shown). In addition, since positive AF[1] and membrane cultures[2,3] are more commonly found in women delivering at or below 30 weeks gestation, outcome by therapy was evaluated in women enrolled at less than 31 weeks. No difference in outcome with antibiotic therapy was found in this subset. Antibiotics had no influence on the pregnancy outcome of patients with markers of infection.

Although antibiotics had no effect on the out-

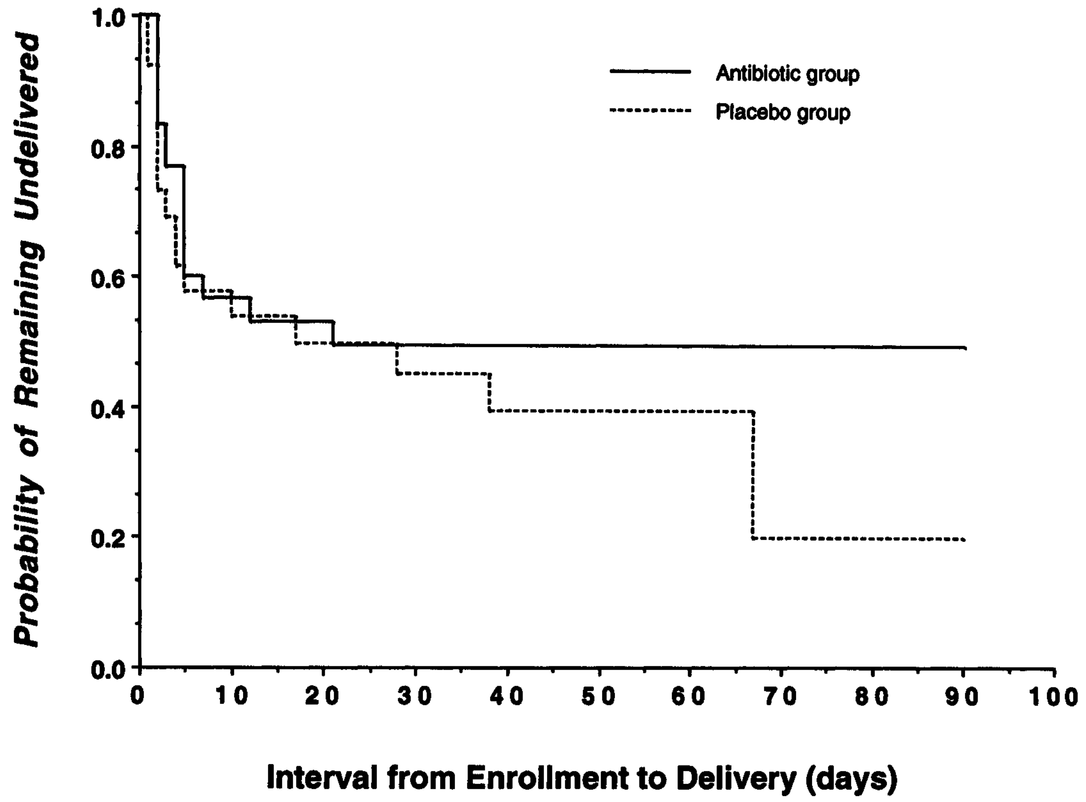

Fig. 1. Probability of remaining undelivered until 35 weeks gestation by randomized treatment assignment.

comes, even among patient groups at higher risk for infection, those patients with markers of infection in either treatment group delivered earlier in gestation. Patients with compared with those without markers of infection (AF WBCs, AF bacteria, and CRP > 1.5 mg/dl) had a significantly shorter interval from enrollment to delivery, a lower gestational age and birth weight at delivery, and longer neonatal hospital stays. The magnitude of difference between those with and without infection or elevated CRP was large enough to represent substantial clinical differences. Patients with compared with those without histologic chorioamnionitis did not have significantly different outcomes.

### DISCUSSION

This study failed to demonstrate any prolongation of pregnancy among antibiotic-treated compared with placebo-treated women. Sample size calculations demonstrate that with the current numbers, this study has greater than 80% power to detect an increase in the proportion of women remaining undelivered for more than 1 week from 50% in the placebo group to 80% in the antibiotic group or a doubling of the interval from enrollment to delivery in the antibiotic group. Thus, it is unlikely that we have failed to detect a significant benefit in the antibiotic group.

Previous studies of antibiotic use among women with PTL with intact membranes have yielded variable results. The previous study most similar to this study evaluated IV ampicillin and oral erythromycin vs. placebo, and no difference in outcome occurred between the 2 groups.[12] Limited evaluations for possible upper genital tract infection were done in that study, but the proportion of women with upper genital tract infection was most likely low because of a mean 34-day prolongation of pregnancy in both groups. This interval was similar to the mean prolongation of pregnancy in patients in PTL without AF bacteria in this and 2 previous studies.[1,13] However, prolongation of pregnancy was found among women in PTL treated with clindamycin compared with placebo, especially among those presenting before 33 weeks gestation.[14] Prolongation of pregnancy also occurred in an unblinded study comparing either oral erythromycin or oral ampicillin with no therapy among women in PTL.[15] In that study, 24% of patients had cervical *C. trachomatis* and 21% of patients had vaginal group B *Streptococcus*, rates of infection much higher than in the present study. Treatment of these infections may have accounted for the observed effect. Additionally, not all of the outcome data in that study appeared normally distributed and more accurate analysis would have been possible using non-parametric statistics.[15] In 2 other reports, oral erythromycin vs. placebo in addition to tocolysis given to women in PTL failed to show any overall prolongation of pregnancy although some effect was demonstrated in those with cervical dilatation of at least 1 cm.[16,17]

The lack of benefit of antibiotic therapy may be related to several factors. First, many patients enrolled had no infection and would not be expected to benefit from antibiotics. Only 14% of those tested had AF infection, and among women not receiving antibiotics before delivery, only 41% had chorioamnion infection and 65% had chorioamnion inflammation. Fifty-five percent of our patients were above 30 weeks gestation at enrollment, a group with only an 11% rate of AF infection and a low rate of membrane infection.[2,3] Thus, the inclusion of women without upper genital tract infection into either group would obscure any potential benefit from antibiotic therapy. Improved tests to identify women at high risk for both AF and membrane infection before delivery are needed to allow targeting of antibiotic trials to assess better the impact on upper genital tract infection.

Some women with upper genital tract infection could have labor that is too advanced at presentation to be inhibited by the antibiotic-tocolytic therapy used in this study. Magnesium sulfate and betamimetic agents were the primary tocolytics. Few patients received prostaglandin synthetase inhibitors. Elevated levels of prostaglandins in the AF have been found among women in PTL with AF infection.[18,19] A combination of antibiotics and prostaglandin synthetase inhibitors might more effectively inhibit PTL than antibiotic-tocolytic agents in patients with upper genital tract infection. However, in one report, pregnancy outcome was not influenced by a combination of antibiotics and indomethacin.[20]

Antibiotics given may fail to eliminate infection at a site critical to the stimulation of labor. A critical site could be the decidua[18] and this area was not sampled after antibiotic therapy in this study, although the rate of positive chorioamnion cultures did not differ between the 2 groups. Antibiotic levels in the decidua and chorioamnion may not be adequate to treat an established infection or may enhance the effects of infection on inciting PTL. Cytokines are released in response to AF infection[18,19] and histologic chorioamnionitis,[19] presumably stimulated by bacterial products. Lysis of bacteria cell walls by antibiotics and release of lipopolysaccharide might even aggravate PTL by further stimulating the cytokine response. Successful attempts to inhibit infected patients in PTL may require the down regulation of the cytokine response before antibiotic therapy by the use of corticosteroids or cytokine antagonists.[21]

It was apparent that the markers evaluated (AF-WBC's, AF bacteria, CRP $> 1.5$ μg/dl) identify patients at significant risk for delivery at a low gestational age and for prolonged infant hospitalization. These markers identify a high-risk group with either infection, inflammation, or some other unidentified upper genital tract condition. Thus, despite the failure of antibiotics to prolong the gestation of patients in PTL, we reject the notion that infection is not related to preterm delivery. Further studies are necessary to prove a causal relationship between the two, and should be directed at the use of rapid tests to detect AF and chorioamnion infection and the evaluation of antibiotic therapy in combination with other therapies such as prostaglandin synthetase inhibitors and cytokine antagonists. In addition, studies are needed to evaluate whether women at increased risk of preterm delivery with upper genital tract infection can be identified and treated earlier in pregnancy to prevent PTL.

### ACKNOWLEDGMENTS

This work was supported in part by a grant from Miles Pharmaceutical Co., Inc.

### REFERENCES

1. Watts DH, Krohn MA, Hillier SL, Eschenbach DA: The association of amniotic fluid infection with gestational age and neonatal outcome among women in preterm labor. Obstet Gynecol 79:351–357, 1992.
2. Hillier SL, Martius J, Krohn M, Kiviat N, Holmes KK, Eschenbach DA: A case-control study of chorioamnionic infection and histologic chorioamnionitis in prematurity. N Engl J Med 319:972–978, 1988.

3. Russell P: Inflammatory lesions of the human placenta. I. Clinical significance of acute chorioamnionitis. Am J Diagn Gynecol Obstet 1:127–137, 1979.
4. Martius J, Krohn MA, Hillier SL, Stamm WE, Holmes KK, Eschenbach DA: Relationships of vaginal *Lactobacillus* species, cervical *Chlamydia trachomatis*, and bacterial vaginosis to preterm birth. Obstet Gynecol 71:89–95, 1988.
5. Stamm WE, Tam MR, Koester M, Cles L: Detection of *Chlamydia trachomatis* inclusions in McCoy cell cultures with fluorescein-conjugated monoclonal antibodies. J Clin Microbiol 17:666–668, 1983.
6. Nugent RP, Krohn MA, Hillier SL: Reliability of diagnosing bacterial vaginosis is improved by a standardized method of Gram stain interpretation. J Clin Microbiol 29:297–301, 1991.
7. Hillier SL, Krohn MA, Kiviat NB, Watts DH, Eschenbach DA: Microbiologic causes and neonatal outcomes associated with chorioamnion infection. Am J Obstet Gynecol 165:955–961, 1991.
8. Siegel S: Nonparametric Statistics for the Behavioral Sciences. New York: McGraw-Hill, pp 111–116, 1956.
9. Cox DR: Regression models and life-tables (with discussion). J R Stat Soc B 34:187–200, 1972.
10. Kaplan EL, Meier P: Nonparametric estimation from incomplete observations. J Am Stat Assoc 53:457–481, 1958.
11. Watts DH, Krohn MA, Wener MH, Eschenbach DA: C-reactive protein in normal pregnancy. Obstet Gynecol 77:176–180, 1991.
12. Newton ER, Dinsmoor MJ, Gibbs RS: A randomized, blinded, placebo-controlled trial of antibiotics in idiopathic preterm labor. Obstet Gynecol 74:562–566, 1989.
13. Gravett MG, Hummel D, Eschenbach DA, Holmes KK: Preterm labor associated with subclinical amniotic fluid infection and bacterial vaginosis. Obstet Gynecol 67:229–237, 1986.
14. McGregor JA, French JI, Kyung S: Adjunctive clindamycin therapy for preterm labor: Results of a double-blind, placebo-controlled trial. Am J Obstet Gynecol 165: 867–875, 1991.
15. Morales WJ, Angel JL, O'Brien WF, Knuppel RA, Finazzo M: A randomized study of antibiotic therapy in idiopathic preterm labor. Obstet Gynecol 72:829–832, 1988.
16. McGregor JA, French JI, Reller LB, Todd JK, Makowski EL: Adjunctive erythromycin treatment for idiopathic preterm labor: Results of a randomized, double-blinded, placebo-controlled trial. Am J Obstet Gynecol 154:98–103, 1986.
17. Winkler M, Baumann L, Ruckhaberle KE, Schiller EM: Erythromycin therapy for subclinical intrauterine infections in threatened preterm delivery—A preliminary report. J Perinat Med 16:253–256, 1988.
18. Romero R, Brody DT, Oyaryum E, Major M, Woy K, Hobbins JC, Durum SK: Infection and labor. III. Interleukin-1: A signal for the onset of parturition. Am J Obstet Gynecol 160:1117–1123, 1989.
19. Hillier SL, Witkin SS, Krohn MA, Watts DH, Kiviat NB, Eschenbach DA: The relationship of amniotic fluid cytokines and prostaglandin $E_2$ with preterm delivery, amniotic fluid infection, histologic chorioamnionitis, and chorioamnion infection. Obstet Gynecol 81:941–948, 1993.
20. Newton ER, Shields L, Ridgway LE III, Berkus MD, Elliott BD: Combination antibiotics and indomethacin in idiopathic preterm labor: A randomized double-blind clinical trial. Am J Obstet Gynecol 165:1753–1759, 1991.
21. Romero R, Tartabzovsky B: The natural interleukin-1 receptor antagonist prevents interleukin-1-induced preterm delivery in mice. Am J Obstet Gynecol 167:1041–1045, 1992.
